# Theoretical Analysis of the Optical Propagation Characteristics in a Fiber-Optic Surface Plasmon Resonance Sensor

**DOI:** 10.3390/s130607443

**Published:** 2013-06-07

**Authors:** Linlin Liu, Jun Yang, Zhong Yang, Xiaoping Wan, Ning Hu, Xiaolin Zheng

**Affiliations:** 1 Key Laboratory of Biorheological Science and Technology, Chongqing University, Ministry of Education, and Key Laboratory of Vision Loss, Regeneration and Restoration, Chongqing, Bioengineering College, Chongqing University, Chongqing 400030, China; E-Mails: liulinlin0103@163.com (L.L.); bmewan@gmail.com (X.W.); zxl@cqu.edu.cn (X.Z.); 2 College of Pharmacy, Third Military Medical University, Chongqing 400038, China; E-Mail: yangzhong1999@yahoo.com

**Keywords:** SPR, fiber-optic, multi-channel, sensor

## Abstract

Surface plasmon resonance (SPR) sensor is widely used for its high precision and real-time analysis. Fiber-optic SPR sensor is easy for miniaturization, so it is commonly used in the development of portable detection equipment. It can also be used for remote, real-time, and online detection. In this study, a wavelength modulation fiber-optic SPR sensor is designed, and theoretical analysis of optical propagation in the optical fiber is also done. Compared with existing methods, both the transmission of a skew ray and the influence of the chromatic dispersion are discussed. The resonance wavelength is calculated at two different cases, in which the chromatic dispersion in the fiber core is considered. According to the simulation results, a novel multi-channel fiber-optic SPR sensor is likewise designed to avoid defaults aroused by the complicated computation of the skew ray as well as the chromatic dispersion. Avoiding the impact of skew ray can do much to improve the precision of this kind of sensor.

## 1. Introduction

With rapid advances of the fabrication technology and transmission theory, optical fiber, which is a medium for long-distance optical transmission, has been extensively used in chemical and biomedical sensing fields [1,2]. During its transmission, light coupled into the optical fiber emerges total internal reflection at the interface of the core and the cladding, which results in negligible optical loss [3]. The fiber core, within which light transmits, can be used as a support of the metal film in the surface plasmon resonance (SPR) detection.

Surface plasmon resonance sensor is the most commonly used optical sensor. In 1902, Wood [4] discovered that there was a loss of a small area of the spectrum after a light beam passed through a grating, and this discovery opened a prelude to the progress of the SPR technology. Until 1971, German physicist Kretschmann [5] used a prism as the substrate and directly covered its bottom with several tens of nanometer thick metal film to achieve SPR detection. Thereafter, SPR technology achieved rapid development. Prism based SPR sensor is widely used in surface analysis, as well as chemical and biological detection. In 1993, Jorgenson and his colleagues [6] succeeded in the SPR detection by using optical fiber as the guide medium of light. They proposed two fiber-based SPR sensor devices and established a viable fiber-optic SPR sensor technology. Then, relative reports rapidly increased in the chemical, biological, environmental, and medicine fields [7–16].

Compared with the conventional prism-based SPR sensor, fiber-optic SPR sensor has many advantages, such as being easy for miniaturization, as well as remote, real-time, and online detection [17]. Owing to the structural particularity of optical fiber, propagation of the light beam within it is exceedingly complex. In previous studies, design of fiber-optic SPR sensors was chiefly dependant on past experiences, and it was difficult to achieve high reliability and accuracy. Besides, chromatic dispersion of light in existing fiber-optic SPR sensors may decrease their detection accuracy. Theoretical analysis of the sensing mechanism and calculation algorithms of all configurations are based on the Maxwell's equation, *i.e.*, the four basic equations of electric and magnetic fields [18,19]. Currently, theoretical analysis of fiber-optic SPR sensors is based on a simplified model [20], where the effect of the propagation of the skew ray, which occupies the most part of the light beam, is not considered. Simultaneous analysis of multiple samples is pursued in more and more biological and chemical analyses, and how to design a simple and reliable optical path for multi-channel fiber-optic SPR sensors is also widely studied [21,22].

In this paper, theoretical analysis of optical propagation, especially the propagation of skew rays, in an optical fiber is undertaken. The resonance wavelength is calculated at two different cases, in which the light dispersion in the fiber core is considered. According to the theoretical analysis, a novel wavelength modulation multi-channel fiber-optic SPR sensor is proposed. This novel design cannot only overcome the defect of optical chromatic dispersion, but also simplify the calculation of the optical path. It can also enhance the sensitivity by eliminating the impact of skew rays, which are neglected in most existing theoretical computation. Thus, high-accuracy multi-channel fiber-optic SPR sensor may be achieved.

## 2. Light Propagation Model in an Optical Fiber

In order to generate a surface plasmon wave in a metal dielectric system, external electromagnetic wave must be irradiated from a transparent dielectric medium to its surface, and the polarization plane of this electromagnetic wave must be overlapped with the incident plane. Thus, only the *p*-polarized light can produce SPR phenomenon [20]. Owing to the characteristic construction of an optical fiber, the metal/dielectric interface of a fiber-optic SPR sensor is no longer a plane instead of a cylindrical surface.

According to different transmitting modes of light, optical fibers can be divided into single mode and multi mode. The center glass core of the single-mode fiber is thin for only a mode of light transmitting, and that of the multi-mode fiber is thick enough to transmit multiple modes of light. Regardless of the mode of optical fiber, a part of the incident light transfers within it is in transverse magnetic (TM) wave form and another part in transverse electric (TE) wave form. Thus, natural light can be used for the fiber-optic SPR sensor, and the separation of the *p*-polarized light and the *s*-polarized light is no longer required [13].

Optical fibers can also be classified as step-index and graded-index according to the distribution of the refractive index. In the step optical fiber, the refractive index from the core to the cladding is abruptly changed. And in the graded fiber, the change of the index is gradual. In this study, the fiber-optic SPR sensor is designed as wavelength modulation and the light source generates compound light, so multimode, step-index optical fiber is chosen.

**Figure 1.**
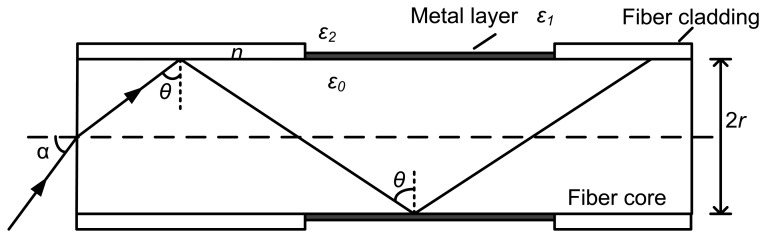
Light transmission mode in an optical fiber.

If an incident light is coupled into a fiber, where its end face is perpendicular to its axis, with an incident angle *α* (Figure 1), the incident angle is *θ* (*θ* > *θ_c_*, *θ_c_* is the critical angle for total reflection) at the interface between the fiber core and cladding. In this case, *ε*_0_, *ε*_1_ and *ε*_2_ are the dielectric constants of the fiber core, metal film, and environmental medium, respectively.

**Figure 2.**
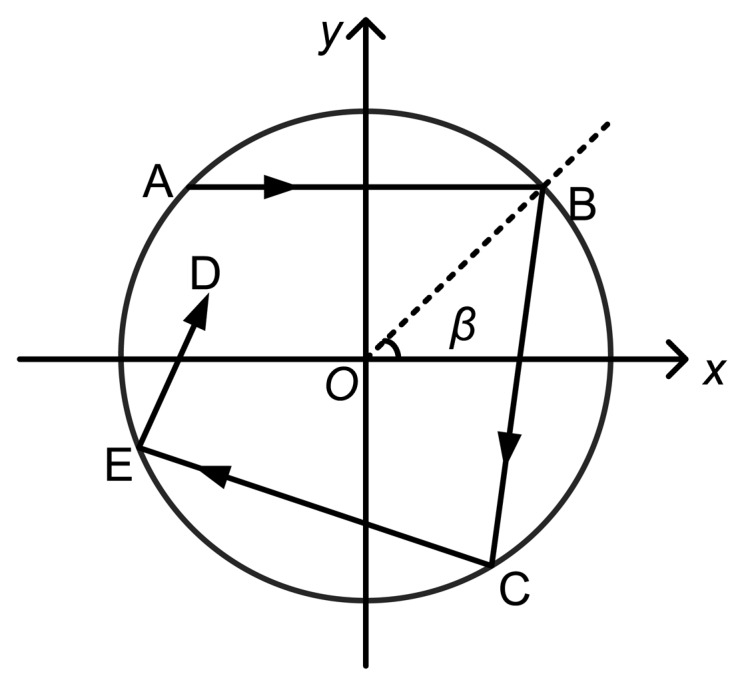
Projection of the propagation path of a skew ray on the cross section of an optical fiber.

The incident light can be separated into a meridional ray and a skew ray. The meridional ray is the component in the meridional plane (the axis of the optical fiber is also on this plane), and the skew ray does not go through the fiber axis but goes forward in a helix form (Figure 2). In most cases, the energy of the incident light is mostly converged within the skew light, and the meridional ray is also regarded as a unique skew light, so only the transmission of the skew ray is discussed in this study.

**Figure 3.**
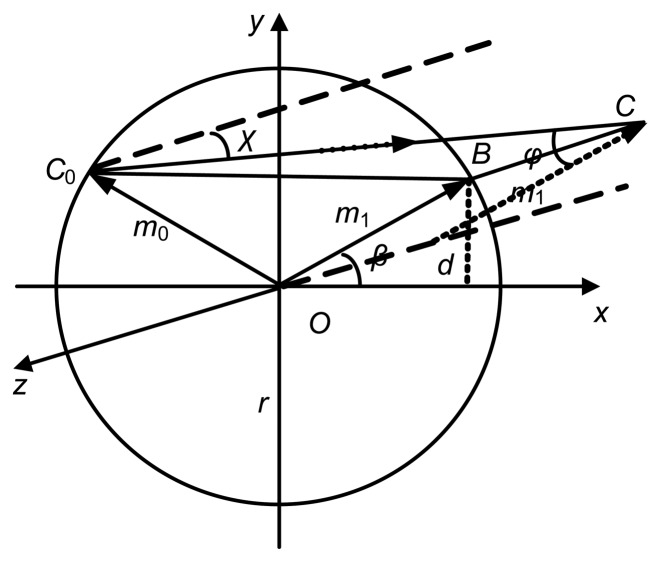
Skew rays of two successive reflections.

Figure 3 is a schematic diagram of the skew ray *C*_0_*C* between two successive total internal reflections (*C*_0_*C* can be regarded anyone of those rays in the Figure 2). Here, *χ* is the angle between the light *C_0_C* and the central axis of the fiber (*z* axis), *φ* is the incident angle of light at the second total reflection, *m*_0_ and *m*_1_ are normal lines at these two successive total reflections, *β* is the angle between normal line *m*_1_ and *x* axis, and two normal lines is symmetric relative to *y* axis. According to the symmetry of the fiber core and the light reflection, when a light beam transmits in an optical fiber, any two successive total reflections can be equivalent as that in Figure 3 regardless of their location. A definite relationship between the angles *χ*, *φ* and *β* is given by [23]:

(1) cosφ=sinχcosβ

In the Figure 3, *BC*_0_ is the projection of the skew ray *C*_0_*C* on the plane *xOy* (cross section). In the *BCC*_0_ plane, light beams parallel with *C*_0_*C* can be equivalent to that in Figure 3. According to the reversibility of light, it can be concluded that all light beams on the incident plane (plane *BOC*_0_) have the same modes when distances (*d*) between the incident points (*C*) and the *x* axis are equal, *i.e.,* they have the same incident angle (*φ*) when these light beams transmit in the total reflective style. The propagation of the skew rays is complicated and it is very difficult to be calculated [24].

## 3. Simulation of the Resonance Wavelength

As the optical fiber structure in Figure 1, light beam is coupled into the fiber with the incident angle *α.* The incident angle is *θ* at the interface between the fiber core and cladding. As previously described, the propagation of skew rays is complicated and it is very difficult to be calculated. The optical fiber SPR sensor can be further simplified to assume that the incident light propagates along a meridian plane without any skew rays in an ideal condition [25]. There is only one total internal reflection at the sensitive film of the entire core. According to the Fresnel formula [26], the reflective coefficient *r* of the sensitive film surface is given by:

(2) $$r=\frac{r_{0,1}+r_{1,2}e^{2ik_{z1^{d}}}}{1+r_{0,1}r_{1,2}e^{2ik_{z1^{d}}}}$$

where *r*_0,1_ is the reflectivity of the interface between the fiber core and metal, and *r*_1,2_ is the reflectivity of the interface between the metal and the ambient medium, *d* is the thickness of the metal layer, *k_z_*_1_ and *k_z_*_2_ are the wave vectors in the *z* direction in the metal layer with refractive index *n*_1_ and ambient medium with refractive index *n*_2_, respectively.

(3) $$r_{0,1}=\frac{\frac{{ɛ}_{0}}{k_{z0}}-\frac{{ɛ}_{1}}{k_{z1}}}{\frac{{ɛ}_{0}}{k_{z0}}+\frac{{ɛ}_{1}}{k_{z1}}}\;\text{and}\;r_{1,2}=\frac{\frac{{ɛ}_{1}}{k_{z1}}-\frac{{ɛ}_{2}}{k_{z2}}}{\frac{{ɛ}_{1}}{k_{z1}}+\frac{{ɛ}_{2}}{k_{z2}}}$$

In the fiber core with refractive index *n*_0_, *k_x_*_0_ and *k_z_*_0_ are the wave vector of the *x* and *z* direction, respectively.

(4) $$k_{x0}=\frac{2\pi }{\lambda }\sqrt{{ɛ}_{0}}\sin\theta \;\text{and}\;k_{z0}=\sqrt{{\left(\frac{2\pi }{\lambda }\right)}^{2}{ɛ}_{0}-{k_{x0}}^{2}}$$

(5) $$k_{z1}=\sqrt{{\left(\frac{2\pi }{\lambda }\right)}^{2}{ɛ}_{1}-{k_{x0}}^{2}}\;\text{and}\;k_{z2}=\sqrt{{\left(\frac{2\pi }{\lambda }\right)}^{2}{ɛ}_{2}-{k_{x0}}^{2}}$$

where *λ* is the wavelength of the light.

A commonly used silica-core multimode fiber is chosen as the sample for the simulation. The diameter of the fiber core (2*r*) is 600 μm, and its refractive index (*n*_0_) is 1.458. The numerical aperture of the fiber (*NA*) is 1.37, and the refractive index of the ambient medium (*n*_2_) is supposed as water (1.3303). Silver film with 50 nm thickness, which is also commonly used in SPR sensors, is coated on the restricted area of the optical fiber as the sensitive layer. Chromatic dispersion *e_s_* of silver is given by [27]

(6) $$e_{s}=\left(\frac{-5.1596}{10^{5}}\times \lambda^{2}-0.0033253\times \lambda +56.2403\right)+j\times \left(\frac{3.1776}{10^{6}}\times \lambda^{2}-0.002018\times \lambda +0.54037\right)$$

According to the equation of the numerical aperture (*NA*)

(7) $$NA={n_{0}}^{2}-n^{2}$$

where *n*_0_ is the refractive index of the fiber core, *n* is the refractive index of the fiber cladding. The refractive index of the cladding (*n*) can be calculated, and the critical angle (*θ_cr_*) of total reflection can also be achieved (about 65.3°) [27].

(8) $$\theta_{\text{cr}}=\sin^{-1}\left(\frac{n}{n_{0}}\right)$$

**Figure 4.**
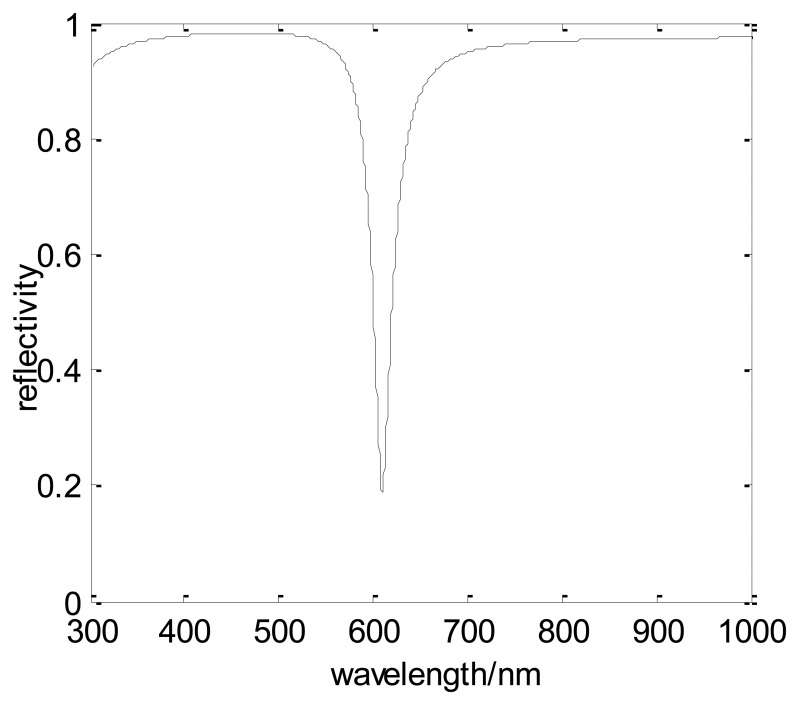
Simulation spectrum of an SPR sensor based on a silica-core multimode optical fiber.

In order to transmit in the optical fiber, the incident angle of the light beam is chosen as 66°. In this model, one end surface of the fiber is cut to an inclined plane (as the following figure) and the angle between this plane and the axis of the fiber is 66°. The incident light enters the optical fiber in a way vertical to the end surface. When the wavelength is changed from 300 nm to 1,000 nm, the simulation result of the SPR curve (interrelationship between the reflectivity and the incident wavelength) can be obtained (Figure 4). In the simulation, the resonance wavelength is about 610 nm. By using this way, the resonance wavelength under a certain condition can be achieved and it will help the design of this kind of fiber-optic SPR sensor. However, in order to simplify the computation, the chromatic dispersion of the fiber core is ignored.

## 4. Design of a Multi-Channel Fiber-Optic SPR Sensor

Light propagates in the optical fiber in two distinct ways, *i.e.*, a meridional ray and a skew ray. The meridional ray always transmits on the same plane, but the skew ray will enter another plane after each total internal reflection, and the trajectory of its optical path is a spatially spiral curve. In order to avoid the complex computation of the skew ray, simulation of the light transmission in optical fiber has been simplified in most previous studies, in which merely the transmission of the meridional ray is considered.

In addition, chromatic dispersion of light is one of the main sources of the experimental error in fiber-optic SPR sensor. When a light beam is coupled into and propagates in an optical fiber, it is affected by various chromatic dispersions, including the dispersion induced by the refraction during the incidence, and the inter-modal, material, and waveguide dispersions during the propagation. Short quartz optical fiber with a large diameter is usually used in fiber-optic SPR sensor, and the dispersion induced by the propagation may be neglected. The dispersion error is mainly caused by the refraction of the incident light of different wavelengths. Owing to its larger diameter, the refractive index of quartz glass is not a proper constant, and it has discrete values for different wavelengths. Natural light used in this kind of fiber-optic SPR sensor is not an ideal light source with a single wavelength, and it has a certain spectral width. When the natural light propagates in the medium of refractive index *n*, the relationship between its transmitting velocity *v* and velocity of light in vacuum *c* is

(9) v=c/n

For the light beams of different wavelengths, they have different refractive indexes and velocities. When a light beam with a certain spectral width transmits within the fiber, diverse components in this compound light have discrete velocity because they have different wavelengths. Thus, there will be a time difference when different components reach the terminal, which will broaden the waveform of the light to generate chromatic dispersion.

If the chromatic dispersion of the fiber core is considered, the resonance wavelength of the fiber-optic SPR sensor can also be calculated. The dispersion of the fiber core *e* is given by [3]

(10) $$e=\frac{1.1399}{10^{10}}\times \lambda^{3}+\frac{2.9294}{10^{7}}\times \lambda^{2}-\frac{2.6420}{10^{4}}\times 10^{4}+1.5356$$

**Figure 5.**
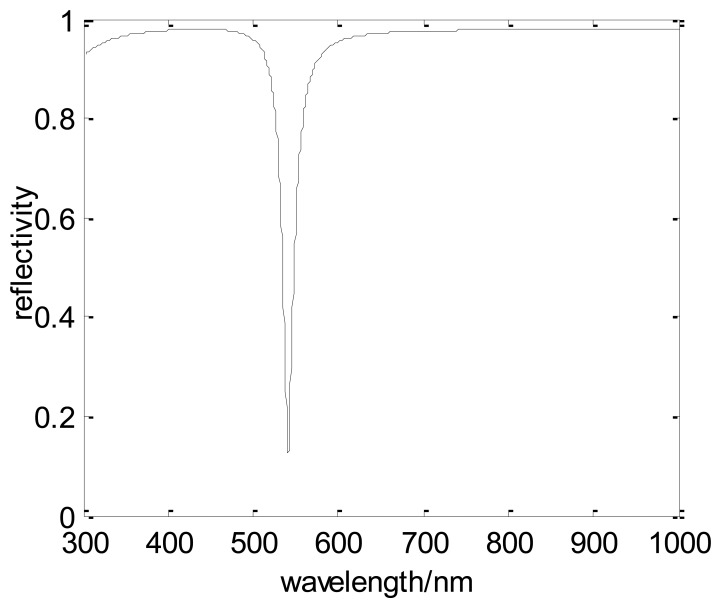
Simulation spectrum of the fiber-optic SPR sensor when the chromatic dispersion of the fiber core is considered.

**Figure 6.**
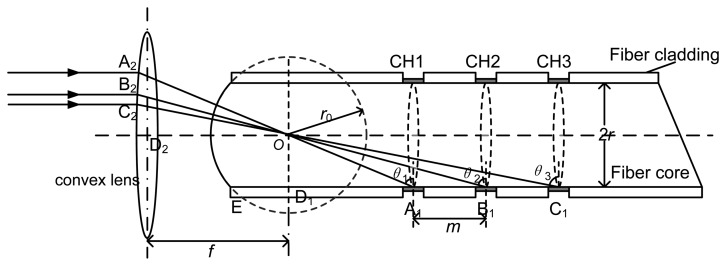
Proposed design of a novel multi-channel fiber-optic SPR sensor.

In this case, the resonance wavelength is 541 nm and the simulation SPR curve is shown in Figure 5. There is an obvious difference between the resonance wavelength whether the dispersion of the fiber core is considered or not. Thus, in the fiber-optic SPR sensor design, the impact of dispersion cannot be ignored. However, the computation is rather complicated when the skew ray and chromatic dispersion are considered at the same time. Thus, an innovative design (Figure 6) is proposed to evade the influence of the skew ray and chromatic dispersion.

Single, dual, or multiple channels can be adopted in this novel fiber-optic SPR sensor. A fiber-optic SPR sensor with three sensitive surfaces (three channels) is used as an example. One exposed end of the fiber core is fabricated as a spherical surface of radius *r*_0_. Incident light beams parallel to the optical axis of a convex lens of focal length f pass through the focal point due to the converging action. Different light beams fall on the three detection regions (A_1_, B_1_, C_1_) of the optical fiber. At these detection regions, sheath of the step-index multi-mode optical fiber was removed by using some etchants such as sulfuric acid, and metal (Ag or Au) film is deposited on the exposed core by rotating the optical fiber in the metal vapor in a high vacuum condition. Then, some ligands are coated on the surface of the metal film. When sample solution flows above the surface of the metal film, target molecules may be bound to the ligands and generate a detectable signal.

The centre of the sphere is located at the axis of the fiber, and the distance between any two adjacent channels is *m.*

For these three different sensitive surfaces, the incident angles are *θ*_1_, *θ*_2_ and *θ*_3_ respectively. These angles can be obtained by

(11) $$\{\begin{array}{c} \tan\theta_{3}-\tan\theta_{2}=\frac{m}{r}\\ \tan\theta_{2}-\tan\theta_{1}=\frac{m}{r}\\ A_{1}E=\sqrt{r_{0}^{2}-r^{2}}+r\tan\theta_{1} \end{array}$$

In sensor design and fabrication, an appropriate *θ*_1_ is chosen (*θ*_1_ > *θ_c_*). Before fabrication of the sensitive surfaces, fiber cladding at A_1_, B_1_ and C_1_ will be taken off and the uncovered surfaces of the core will be cleaned with an appropriate solvent. Usually, gold or silver film will be deposited on the uncovered surface of the core to form sensitive surfaces. A convex lens of focal length *f* will be placed in front of the circular-arc-shaped end of the optical fiber, and its focus must be superposed at the center of the sphere. The distances between different incident points on the lens (A_2_, B_2_, C_2_) and the central axis can be given by

(12) $$A_{2}D_{2}=\frac{f}{\tan\theta_{1}},B_{2}D_{2}=\frac{f}{\tan\theta_{2}},C_{2}D_{2}=\frac{f}{\tan\theta_{3}}$$

In most traditional fiber-optic SPR sensor [9], an optical fiber with an end face perpendicular to its axis is utilized, and an incident light is coupled into the fiber core directly. The impact of skew ray and chromatic dispersion cannot be avoided. In contrast, the front end of the optical fiber used in this new design is processed as a spherical surface and a convex lens is used to couple the incident light. As a consequence, the incident light is perpendicular to the end surface of the optical fiber, and there is no chromatic dispersion occurred at the interface (the spherical face with centre *O* in Figure 6) of two different mediums. For any sensing points (A1, B1, C1 in Figure 6), chromatic dispersion can be avoided by chosen suitable incident points (A2, B2, C2) on the end surface of the optical fiber. However, incident angles for any sensing region include a range because this region cannot be regarded as an ideal point. Thus, these sensing regions must be fabricated as short as possible to reduce the range of incident angles which can cover the entire sensing areas. Theoretically, this innovative design can overcome the influence of the chromatic dispersion. Furthermore, because the incident light and the axis of the optical fiber are always on the same plane, there is no skew ray, and the complicated computation of the propagation of the skew ray is similarly not required. In order to improve the sensitivity of fiber-optic SPR sensor, most existing theoretical analysis and experimental methods are tried to change the shape of sensing region (e.g., retro-reflecting or tip tapered) [28], or change some parameters such as numerical aperture (NA), ratio of sensing region length to fiber core diameter, total bimetallic thickness, maximum absorption wavelength, and half maximum width of the sensing medium [29,30]. Compared with those studies, the model in this study is easier for realization because the impact of skew rays is avoided.

## 5. Conclusions

In this study, a theoretical model of the light transmitting within a fiber-optic SPR sensor is set up. It is based on the mature theoretically analytical method of the traditional prism based SPR sensor, and can provide theoretical support for the design of a new fiber-optic SPR sensor. The transmission of a skew ray within an optical fiber is discussed and the influence of the chromatic dispersion of the fiber core is also analyzed. Resonance wavelengths of different cases, in which the dispersion of the fiber core is considered, are simulated. These results show that the skew ray and dispersion have significant impacts on the precision of the sensor, and they are problematic for accurately calculation. In many existing methods and models, computation of the skew ray cannot be avoided in the design, so it is difficult to ensure high detection precision of the fiber-optic SPR sensor. In this study, a novel multi-channel fiber-optic SPR sensor is established, and the complicated computation of the transmission of a skew ray and the chromatic dispersion is not required in this device. Thus, accurate estimation of the resonance wavelength can be achieved.
